# Mitophagy is required to protect against excessive skeletal muscle atrophy following hindlimb immobilization

**DOI:** 10.1186/s12929-025-01118-w

**Published:** 2025-02-18

**Authors:** Fasih A. Rahman, Mackenzie Q. Graham, Amanda M. Adam, Emma S. Juracic, A. Russell Tupling, Joe Quadrilatero

**Affiliations:** Faculty of Health, Department of Kinesiology and Health Sciences, University of Waterloo, 200 University Ave. West, Waterloo, ON N2L 3G1 Canada

**Keywords:** Apoptosis, BNIP3, Disuse atrophy, Mitophagy, Mitochondria, Skeletal muscle

## Abstract

**Background:**

Skeletal muscle atrophy involves significant remodeling of fibers and is characterized by deficits in mitochondrial content and function. These changes are intimately connected to shifts in mitochondrial turnover, encompassing processes such as mitophagy and mitochondrial biogenesis. However, the role of these mitochondrial turnover processes in muscle atrophy remains poorly understood.

**Methods:**

We used a novel mitophagy reporter model, mt-Keima mice, to perform hindlimb immobilization and accurately measure mitophagy. A comprehensive set of analyses were conducted to investigate biochemical and molecular changes at the muscle and mitochondrial levels. We also performed image analyses to determine mitophagic flux. To further explore the role of mitophagy in immobilization-induced atrophy, we treated animals with N-acetylcysteine (NAC; 150 mg/kg/day) to modify reactive oxygen species (ROS) signaling and colchicine (0.4 mg/kg/day) to inhibit autophagy.

**Results:**

Our study revealed that hindlimb immobilization leads to muscle weakness and atrophy of fast-twitch muscle fibers (types IIA, IIX, and IIB), with recovery observed in IIA fibers following remobilization. This atrophy was accompanied by a significant increase in mitophagic flux. Additionally, immobilization induced notable mitochondrial dysfunction, as shown by diminished respiration, increased mitochondrial ROS, and greater whole muscle lipid peroxidation. Treatment of immobilized mice with NAC enhanced mitochondrial respiration and reduced ROS generation but suppressed mitophagic flux and intensified atrophy of type IIX and IIB fibers. Additionally, administration of colchicine to immobilized mice suppressed mitophagic flux, which also exacerbated atrophy of IIX and IIB fibers. Colchicine treatment led to significant reductions in mitochondrial function, accompanied by CASP9 and CASP3 activation.

**Conclusion:**

These findings emphasize the role of mitophagy in limiting excessive muscle atrophy during immobilization. Targeting mitophagy may offer new strategies to preserve muscle function during prolonged periods of immobilization.

## Introduction

Skeletal muscle atrophy is characterized as a catabolic state that results in the rapid deterioration of muscle mass and impaired muscle function. Several physiological and pathological conditions can result in skeletal muscle atrophy, including aging, denervation, immobilization, neurodegeneration, and myopathy [1–5]. A common feature of these conditions is an imbalance towards greater protein degradation in muscle.

Skeletal muscle atrophy is associated with the activation of the ubiquitin-proteosome system (UPS), apoptosis, and autophagy. UPS is known to degrade myofibrillar proteins (i.e., actin and myosin) during atrophy, while activation of the intrinsic apoptotic signaling cascade may function to eliminate myonuclei or entire fibers [6–9]. Furthermore, the role of autophagy has been implicated in the maintenance of muscle mass [10] and in disuse atrophy [3]. Autophagy functions to degrade damaged cellular material to allow the recycling of macromolecules to build new proteins. Autophagy can occur in a selective or non-selective manner. With respect to skeletal muscle, the selective degradation of mitochondria, termed mitophagy, has been implicated in maintaining normal function of fibers [11]. Dysfunction or dysregulation of mitophagy has been associated with impaired skeletal muscle function [2]. In contrast, activating mitophagy through urolithin A can improve muscle size and function in dystrophic and aged rodents [12, 13], suggesting a potential protective role for mitophagy.

Atrophy of muscle fibers can occur within one week of disuse and is accompanied by elevated mitophagy signaling. Elevated levels of PINK1, PRKN and *Bnip3l* have been reported in immobilized and remobilized skeletal muscle [14, 15]. Similarly, greater *Bnip3, Bnip3l,* and *Prkn* is observed in immobilized skeletal muscle [16] along with elevated mitophagic flux [16]. Consistent with these findings, elevated mitophagy flux occurs as early as 1-day following denervation [3]. Therefore, a clear relationship between mitophagy and disuse atrophy has been established; however, it has yet to be determined whether mitophagy is contributing to the atrophy or if mitophagy is activated as a protective signaling mechanism during atrophy. Thus, the upregulation of mitophagy may be complementary to atrophic conditions, given the lack of energy demand placed on the mitochondria or perhaps due to the intrinsic dysfunction within the mitochondria. The upregulation of mitophagy may serve as an initiating step for atrophy by reducing the bioenergetic status of the fibers; however, further investigation is required to delineate this relationship. Alternatively, mitophagy may play a protective role to prevent excessive cellular damage caused by mitochondrial dysfunction.

The purpose of the present study was to understand the role of mitophagy following immobilization and remobilization of skeletal muscle. We further questioned whether inhibition of ROS via antioxidant treatment or autophagy via colchicine treatment could modify mitophagy and immobilization-induced muscle atrophy.

## Material and methods

### RNA sequencing analysis

Publicly available RNA sequencing datasets were retrieved from the Gene Expression Omnibus (GEO) database (accession number GSE237537). Data from control (CTRL) and immobilized (IM) gastrocnemius muscles were processed for RNA-Seq analysis, as previously described [17]. Initial quality control was based on exclusion criteria including adapter contamination and low-quality bases. Gene-level expression was quantified and analyzed using the DESeq2 package in RStudio. Differential expression was determined with an adjusted p-value of < 0.05 and a minimum log2 fold change of > 1, identifying significantly differentially expressed genes. Enrichment of biological processes and pathways was assessed using the clusterProfiler package, with tests against Gene Ontology (GO) terms. Pathways were considered significantly enriched at a corrected p-value of < 0.05. Visualization of the results, including volcano plots, heatmaps, and barplots, were created using GraphPad PRISM software.

### Animals

Mt-Keima mice were kindly gifted by Torin Finkel [18, 19]. These mice are a valuable tool for studying mitophagy since they express the fluorescent Keima protein conjugated to the COX8 subunit found within complex IV in the inner mitochondrial membrane. The unique feature of the ratiometric Keima molecule is its dual-excitation, single-emission fluorescence. Under physiological pH, as found in the cytosol, the shorter wavelength excitation predominates (i.e., 488 nm). In the acidic conditions of the lysosome, where mitochondria are degraded, the excitation gradually shifts to a longer wavelength (i.e., 561 nm). This shift allows for precise monitoring of mitophagy. Additionally, the resistance of this molecule to lysosomal degradation enhances the accuracy of mitophagic flux measurements.

Mice were given free access to food and water. All animal procedures were conducted in accordance with the standards set by the Canadian Council on Animal Care (CCAC) and were approved by the Animal Care Committee (ACC) at the University of Waterloo.

### Hindlimb immobilization and drug treatment

Mice were anesthetized under light isoflurane inhalation (~ 2%). The left hindlimb of each mouse was extended and a small (1 cm x 3 cm) piece of Dermapore tape (3M) was wrapped around the ankle and knee joints. An elastic self-adhesive tape (Tensor; 3 cm x 5 cm) was lightly wrapped on top. One additional layer of medical tape was added on top to secure the self-adhesive tape. All mice regained consciousness after 5 min and signs of discomfort or over-constriction were monitored. In a subset of experiments, vehicle (VEH; saline) or N-acetylcysteine (NAC; 150 mg/kg/day) were injected daily for the duration of the immobilization phase. In another subset of experiments, VEH (saline) or colchicine (COL; 0.4 mg/kg/day) were injected daily for the final 5-days of the immobilization phase.

### Isolation of skeletal muscles

The extensor digitorum longus (EDL), tibialis anterior (TA), gastrocnemius (GAS), and quadriceps (QUAD) muscles were rapidly excised from mice. Intact EDL muscle was immediately transferred to Tyrode buffer (121 mM NaCl, 5 mM KCl, 24 mM NaHCO_3_, 1.8 mM CaCl_2_, 0.4 mM NaH_2_PO_4_, 5.5 mM glucose, 0.1 mM EDTA, and 0.5 mM MgCl_2_, pH 7.3) at room temperature for contractility measurements. One TA was stored in BIOPS buffer (2.77 mM CaK_2_EGTA, 7.23 mM K_2_EGTA, 5.77 mM Na_2_ATP, 6.56 mM MgCl_2_−6H_2_O, 20 mM taurine, 15 mM Na_2_PCr, 20 mM imidazole, 0.5 mM DTT, 50 mM K-MES) on ice until use for respirometry and confocal imaging. The contralateral TA muscles were embedded in OCT compound (Tissue Tek), frozen in isopentane cooled by liquid nitrogen, and stored at − 80 °C for fiber type analysis. The GAS and QUAD muscles were cut in half and snap frozen for immunoblot analysis, while the remaining half was stored in PBS-EDTA solution (10 mM EDTA in 1X PBS, pH 7.4) on ice for mitochondrial fractionation.

### Live myofiber imaging and mitophagy flux analysis

The TA muscle was manually separated into small bundles in BIOPS buffer on ice. Bundles were rapidly transferred to #1 coverslips and carefully separated to allow maximal contact of muscle bundle to the coverslip. A thin layer of 0.8% agarose gel was placed on top of the bundles to prevent movement during imaging. Five to ten fields consisting of 3–5 fibers were imaged on a Zeiss LSM 800 confocal microscope at 63X magnification. Individual excitation wavelengths were used to minimize overlap between fluorescent channels. An excitation wavelength of 488 nm was used to determine healthy mitochondria (pH ~ 7; green), whereas an excitation wavelength of 561 nm was used to determine mitochondria undergoing mitophagy (pH ~ 4; red). Emission for both excitation wavelengths was the same at 620 nm. Puncta count and size were determined in each field using the automated Particle Analysis function in ImageJ.

### High-resolution respirometry with simultaneous ROS measurements

The TA muscle was separated under a stereomicroscope into bundles using very fine tipped forceps in ice-cold BIOPS buffer. Bundles were permeabilized in 50 μg/ml of saponin in 1.5 ml BIOPS buffer for 30 min on ice with gentle rocking. Muscle bundles (1–3 mg) were then transferred to 1.5 ml of buffer Z (105 mM K-MES, 30 mM KCl, 1 mM EGTA, 10 mM K_2_HPO_4_, 5 mM MgCl_2_–6H_2_O, 5 mg/ml BSA, pH 7.1) to wash prior to respirometry experiments. Oxygen consumption and mitochondrial hydrogen peroxide (mH_2_O_2_) emission were determined in buffer Z supplemented with 10 μM blebbistatin, 10 μM Amplex Red, 5U/ml horseradish peroxidase, and 10U/ml superoxide dismutase using an Oxygraph-2 K equipped with a LED2 fluorometry module. Experiments were conducted at 37 °C in a 2 ml chamber that was hyperoxygenated to ~ 350 μM. Reverse electron flow was determined with the addition of 10 mM succinate, followed by 5 mM pyruvate and 2.5 mM malate. Maximal coupled respiration was stimulated with the addition of 5 mM ADP. Cytochrome c was added to ensure outer membrane integrity. mH_2_O_2_ emission was calibrated from a standard curve established with the same reaction conditions.

### Mitochondrial fractionation

GAS and QUAD muscles were chopped on a stainless-steel board using a razor for one minute. The tissue was transferred back into the same tube containing 5 ml of PBS with 10 mM EDTA (PBS-EDTA solution) and centrifuged at 500 g for 5 min. The supernatant was discarded, and the muscles were digested in 0.05% trypsin in PBS-EDTA solution for 5 min on ice. The trypsin solution was removed by centrifugation at 500 g for 5 min. Digested muscles were resuspended in mitochondrial isolation buffer (100 mM KCl, 50 mM MOPS, 5 mM MgSO_4_−7H_2_O, 1 mM EGTA, 2 mg/ml BSA, pH 7.4) and homogenized using a Teflon homogenizer equipped on a drill press set to 200 rpm for 5–10 strokes. The homogenized tissue was subjected to low-speed centrifugation (~ 700 × g) to pellet large debris and the supernatant was transferred to ultracentrifuge tubes and spun at 10,000 g for 15 min at 4 °C. The resulting pellet containing mitochondria was resuspended in mitochondrial isolation buffer without BSA before being transferred to 1.5 ml microcentrifuge tubes and spun at 10,000 g for 5 min. The resulting pellet was washed and centrifuged three times in mitochondrial isolation buffer without BSA to remove any residual cytosolic content. After the final spin, the supernatant was aspirated, and the pellets were frozen in liquid nitrogen as the mitochondrial enriched fraction.

### Immunoblotting

Immunoblot analyses were performed as previously described [20, 21]. Snap frozen muscle samples were divided into 20–30 mg portions and stored in separate tubes. Muscle samples were crushed into a powder using a liquid nitrogen-cooled pestle and mortar and transferred to 1.5 ml tubes containing 5 volumes of lysis buffer (20 mM HEPES, 10 mM NaCl, 1.5 mM MgCl_2_, 1 mM DTT, 20% glycerol, 0.1% Triton-X100, pH 7.4) with protease inhibitor. Mitochondrial enriched fractions were resuspended in lysis buffer with protease inhibitor and sonicated at 40 Hz for 10 s on ice.

Protein content was determined using a BCA protein assay. Equal protein was loaded and separated using 8–12% SDS-PAGE, transferred on to PVDF membranes, and blocked with 2–5% non-fat milk powder in TBS-T at room temperature for 1 h. Membranes were briefly rinsed and incubated overnight in primary antibodies against: BNIP3 (#3769, Cell Signaling), BNIP3L (#12396, Cell Signaling), MAP1LC3B/LC3B (#2775, Cell Signaling), GAPDH (#2118, Cell Signaling), OPA1 (#80471, Cell Signaling), MFN2 (#9482, Cell Signaling), DNM1L (#8570, Cell Signaling), SQSTM1 (PM045, MBL), PINK1 (sc-33796, Santa Cruz), PRKN (sc-32282, Santa Cruz), VDAC1 (sc-390996, Santa Cruz), ANT1 (sc-9299, Santa Cruz), CYCS (sc-13256, Santa Cruz), TFAM (sc-166965, Santa Cruz), BAX (sc-526, Santa Cruz), BCL2 (sc-7382, Santa Cruz), ACT (S2066; Sigma), PPARGC1A (ST1202, Sigma), SOD1 (SOD-101, Enzo Life Sciences), SOD2 (SOD-110, Enzo Life Sciences), and 4HNE (ab46545, Abcam). Following incubation, membranes were washed, and appropriate horseradish peroxidase-conjugated secondary antibody was applied at room temperature for 1 h. Bands were visualized using ECL substrate on a ChemiDoc Imaging System.

### Caspase, calpain, and proteasomal activity assay

To measure caspase (CASP), calpain (CAPN) and proteasomal activity, whole‐muscle lysates were collected as above in the absence of protease inhibitors [22–24]. Samples were incubated in duplicates with 20 μM of Ac‐DEVD‐AFC (AAT Bioquest, 13,401) for CASP3 or Ac‐LEHD‐AFC (Tocris Bioscience, 1575) for CASP9 in assay buffer (20 mM HEPES pH 7.4, 10 mM DTT, and 10% glycerol). For CAPN activity, samples were incubated in duplicates with 20 μM of Suc-LLVY-AMC (Enzo Life Sciences, BML-P802) and Z-LL-CHO (Enzo Life Sciences, BML-PI116) inhibitor in assay buffer for 2 h at 30 °C. For 20S proteasomal activity, samples were incubated in duplicates with 20 μM of Suc-LLVY-AMC and MG-132 (Adooq Bioscience, A11043) inhibitor in proteasome assay buffer (50 mM Tris/HCl, 25 mM KCl, 10 mM NaCl, 1 mM MgCl_2_; pH 7.5) for 1 h at 30 °C. Fluorescence measurements were performed at room temperature using a Cytation 5 Imaging Multi‐Mode Reader. The AFC probe was measured with excitation and emission wavelengths at 400 and 505 nm, respectively. The AMC probe was measured with excitation and emission wavelengths at 360 and 440 nm, respectively. The fluorescence values were normalized to protein concentration of samples and expressed as fold changes in fluorescence.

### Immunofluorescence fiber type analysis

Ten-micron cryosections of TA were cut, mounted onto microscope slides and maintained at −20 °C until staining, as previously described [25, 26]. For staining, slides were dried at room temperature, circumscribed with a hydrophobic pen and blocked using 10% goat serum in PBS. Slides were incubated at room temperature with antibodies against type IIA (SC-71, DSHB), type IIX (6H1, DSHB), and dystrophin (DMD; MANDYS1, DSHB) for 2 h. Slides were washed three times with PBS and incubated at room temperature with appropriate secondary antibodies. Following incubation with secondary antibodies, slides were washed three times and mounted with Fluoromount-G (Thermo Fisher Scientific) mounting medium. Fiber cross-sectional area (CSA) and fiber counts was quantified using ImageJ.

### Skeletal muscle contractility

Intact EDL muscles were placed in a 25 °C bath of oxygenated Tyrode solution, as previously described [27, 28]. Muscles were placed between two platinum electrodes driven by a biphasic stimulator. Muscle stimulation occurred at varying frequencies from 10 to 150 Hz. After the stimulation protocol, a fatigue protocol was performed by stimulating at 70 Hz for 350 ms every 2 s for a total of 5 min. The number of contractions to reduce force by 50% was used as an index of fatigue. Data were analyzed using Dynamic Muscle Control Data Acquisition software (Aurora Scientific). Specifically, peak isometric force amplitudes (mN) were determined across the range of stimulation frequencies and normalized to muscle mass.

### Statistics

Statistical analyses were performed in GraphPad PRISM. T-tests were performed to compare changes in functional parameters between control (CTRL) and immobilized (IM) or remobilized muscle (RM). T-tests were also performed to compare VEH to treatment in immobilized muscle (COL or NAC). An alpha value of 0.05 was considered statistically significant.

## Results

### Immobilization-induced muscle atrophy upregulates cellular stress signaling genes and downregulates mitochondria-related genes

**Fig. 1:**
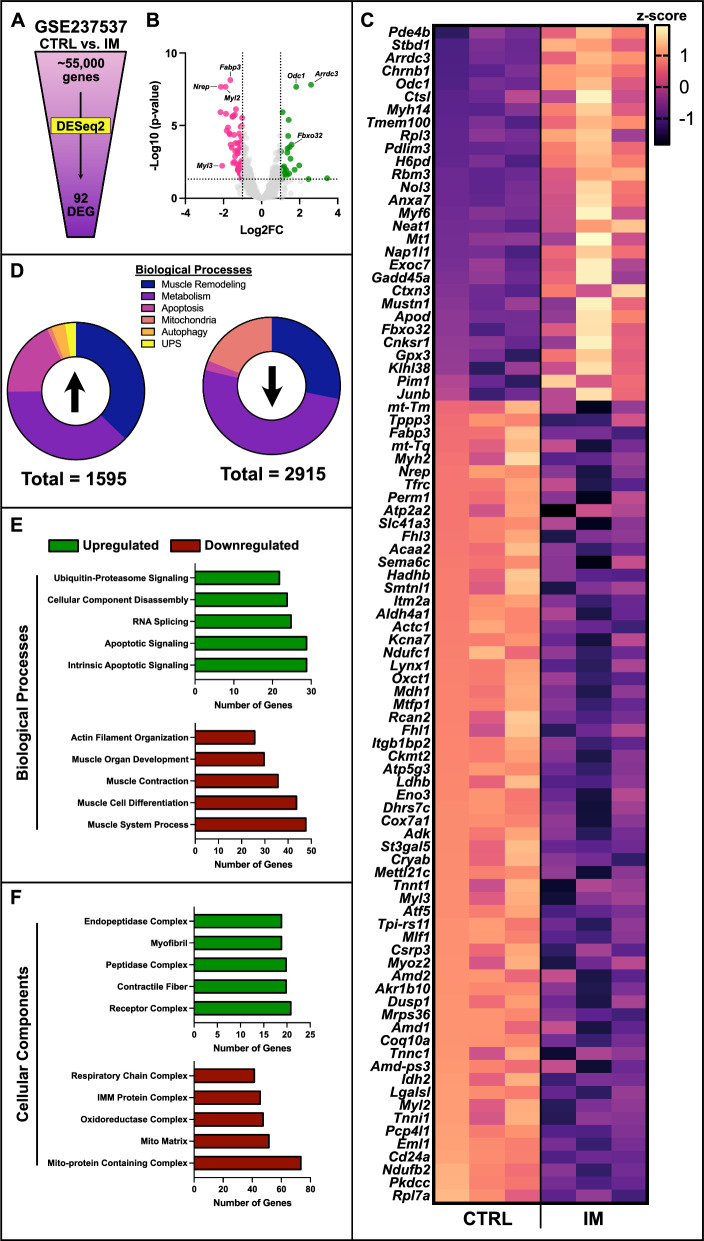
RNA sequencing analysis of control (CTRL) and immobilized (IM) gastrocnemius muscle. **A** Representative visualization of genes analyzed from the publicly available Gene Expression Omnibus database (Accession ID: GSE237537). **B** Volcano plot displaying differential gene expression in skeletal muscle. The horizontal line delineates a significance threshold of p < 0.05. Vertical lines mark a z-score change greater than Log2. Green labels indicate upregulated differentially expressed genes (DEGs), while red labels indicate downregulated DEGs. **C** List of all 92 DEGs identified by DESeq2 analysis. **D** Gene ontology analyses and donut graph outputs summarizing changes in genes associated with five major remodeling processes: muscle remodeling, metabolism, apoptosis, mitochondria, autophagy, and the ubiquitin proteasome system (UPS). A total of 1595 genes related to these processes are upregulated while 2915 genes related to these processes are downregulated. **E** Gene ontology analyses output of the number of genes linked to the top five upregulated (green) and downregulated (red) biological processes. **F** Gene ontology analyses output of the number of genes linked to the top five upregulated (green) and downregulated (red) cellular components. CTRL indicates control mice (i.e., no intervention). IM indicates mice collected following 7-days of immobilization. n = 3 per group

Immobilization-induced muscle atrophy leads to mitochondrial remodeling, affecting density, morphology, and oxidative capacity, which impact muscle integrity and function [29–31]. Changes in proteins related to mitochondrial biogenesis, mitophagy, and dynamics underscore the complex regulation of disuse responses [3, 4, 16, 32, 33]. Understanding these adaptations is key to preserving muscle mass and function during immobilization. To this end, we started with a broad RNA-Seq analysis using a publicly available dataset from the GEO database (GSE237537). From this dataset, approximately 55,000 genes were processed and filtered prior to analysis. Differential expression analysis, conducted using DESeq2, identified 92 differentially expressed genes (DEGs) between the immobilized (IM) and control (CTRL) groups (p < 0.05; Fig. 1A–C). Among these DEGs, several genes associated with cellular stress signaling were upregulated (e.g., *Fbxo32* and *Gadd45a*), while skeletal muscle-related genes were downregulated (e.g., *Myl2* and *Myl3*) in the IM group compared to CTRL (Fig. 1B, C). Notably, no DEGs directly associated with mitochondrial remodeling, particularly in terms of mitochondrial biogenesis, mitophagy, or mitochondrial dynamics were observed.

Gene ontology (GO) analysis revealed significant upregulation in genes related to autophagy and the UPS, with little upregulation of mitochondrial-related genes (Fig. 1D). Detailed analysis of the top upregulated biological processes and cellular components revealed a greater number of genes associated with degradative pathways, including apoptosis and UPS (Fig. 1E, F). Among the major downregulated biological processes and cellular components were genes associated with muscle development/remodeling and mitochondrial function (Fig. 1E, F). Overall, this analysis highlighted significant changes in gene expression profiles due to immobilization, with a notable upregulation of genes associated with degradative processes and a downregulation of genes related to mitochondrial components.

### Hindlimb immobilization leads to greater mitochondrial ROS emission, increased mitophagic flux, and atrophy of all fibers

**Fig. 2:**
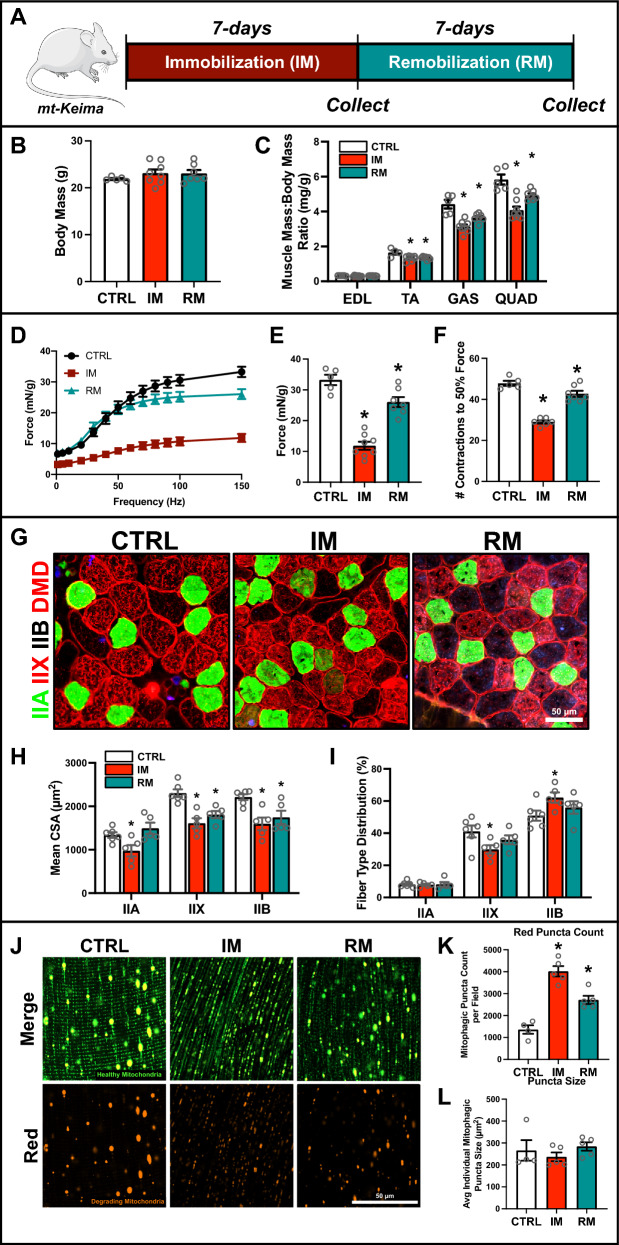
Changes in skeletal muscle structure and mitophagic flux in IM and RM muscle. **A** Representative visualization of immobilization and remobilization protocol. **B** Body mass of control (CTRL), immobilized (IM), and remobilized (RM) mice. **C** Skeletal muscle mass of tibialis anterior (TA), extensor digitorum longus (EDL), gastrocnemius (GAS), and quadriceps (QUAD) normalized to body mass. **D** Muscle force-frequency graph from CTRL, IM, and RM mice. **E** Peak force development derived from force-frequency curve and **F** fatigue index measurement of EDL (greater value indicates more fatigue-resistance). **G** Representative immunofluorescent images of muscle from CTRL, IM, and RM mice. Samples were stained to identify myosin heavy chain isoform and fiber membrane: type IIA (green), type IIX (red), type IIB (unstained), and dystrophin (DMD, red). Scale bar indicates 50 μm. **H** Quantification of mean cross-sectional area (CSA) and **I** distribution of type IIA, IIX, and IIB fibers. **J** Representative confocal images of mitophagic flux from muscle of mt-Keima reporter mice. Green fluorescence indicates healthy mitochondria. Red fluorescence indicates degrading mitochondria. Scale bar indicates 50 μm. Quantification of **K** mitophagic (i.e., red) puncta count per field and **L** average individual mitophagic puncta size. * p < 0.05 compared to CTRL group. n = 5–8 per group

**Fig. 3:**
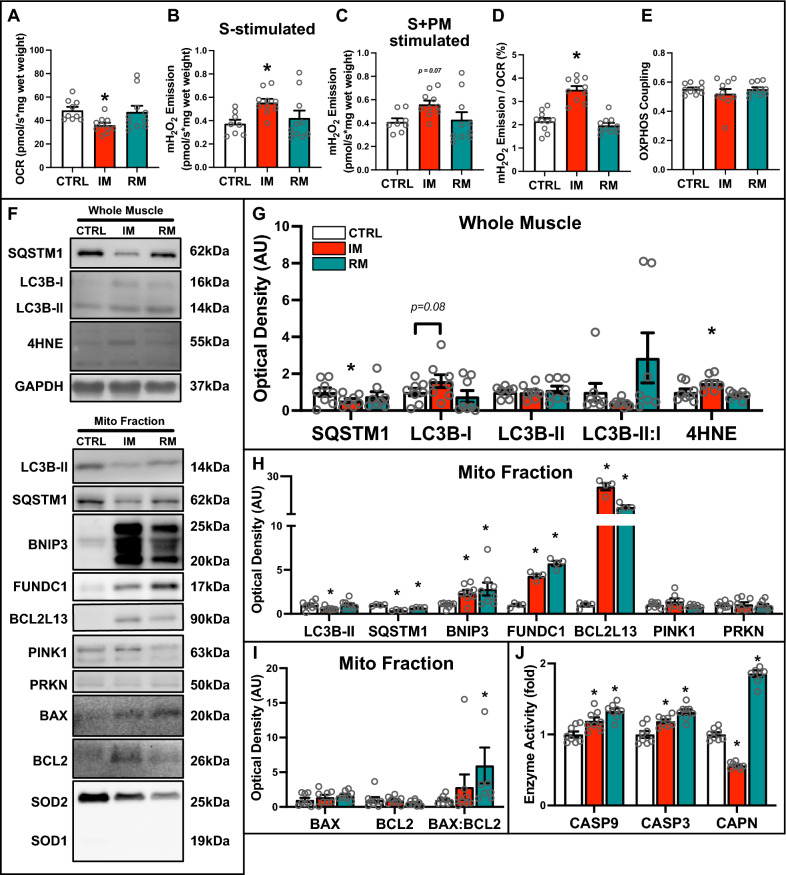
Quantitative analysis of autophagy and mitophagy proteins in IM and RM muscle. **A** Quantification of maximal oxygen consumption rate (i.e., respiration) of permeabilized bundles. Simultaneous quantification of **B** succinate-stimulated mitochondrial hydrogen peroxide (mH_2_O_2_) production, and **C** succinate + pyruvate/malate-stimulated mH_2_O_2_ production in permeabilized muscle bundles. **D** Quantification of mitochondrial fraction of electron leak and **E** oxidative phosphorylation (OXPHOS) coupling. **F** Representative immunoblots of whole muscle lysates and mitochondrial fractions. GAPDH shown as a loading control for whole muscle lysates. SOD1 (cytosolic) and SOD2 (mitochondrial) shown to confirm purity of mitochondrial fraction. **G** Quantitative analysis of SQSTM1, LC3B-I, LC3B-II, LC3B-II:I, and 4HNE from whole muscle lysate. **H** Quantitative analysis of LC3B-II, SQSTM1, BNIP3, FUNDC1, BCL2L13, PINK1, and PRKN from mitochondrial-enriched fractions. **I** Quantitative analysis of BAX, BCL2, and BAX:BCL2 from mitochondrial-enriched fractions. **J** Quantitative analysis of CASP9 activity, CASP3 activity, and CAPN activity. * p < 0.05 compared to CTRL group. n = 6–8 per group

To further our understanding, we investigated physiological adaptations in muscle using a hindlimb bandage model for 7-days of immobilization (IM) in order to induce atrophy, followed by a 7-day remobilization (RM) period to assess recovery dynamics in mt-Keima reporter mice (Fig. 2A). Our findings indicated that IM led to approximately 20% decrease in the relative muscle mass of major hindlimb muscles including the tibialis anterior (TA), gastrocnemius (GAS), and quadriceps (QUAD), which persisted even after 7-days of RM with no change in body mass (Fig. 2B, C). Functional assessments revealed a significant decrease in muscle force and greater fatiguability in both IM (−66% and −40%, respectively) and RM (−22% and −12%, respectively) EDL compared to CTRL (Fig. 2D–F). RM muscle showed improvements in muscle function following IM, achieving 78% recovery of muscle force and 89% recovery of fatiguability compared to CTRL (Fig. 2D–F). Detailed fiber type analysis revealed a specific decline in the CSA of type IIA fibers during the IM phase (− 27%; p < 0.05), which normalized upon RM. However, the mean CSA of type IIX and IIB fibers remained reduced (− 22 to − 28%; p < 0.05) in IM and RM phases (Fig. 2G, H), indicating a fiber type-specific susceptibility to IM, particularly affecting fibers with a faster more glycolytic phenotype. Furthermore, we observed a shift in fiber type distribution, with an increase in type IIB fibers and a decrease in type IIX fibers during IM, which recovered after remobilization (Fig. 2I). Utilizing the mt-Keima reporter mice we were able to investigate mitochondrial quality and mitophagic flux. We found a significant increase in mitophagic puncta count in IM (threefold) and RM (twofold) muscle compared to CTRL; albeit no changes in average mitophagic puncta size (Fig. 2J–L). This indicates that immobilization-induced muscle atrophy is closely linked to the activation of mitophagy. Mitophagy is upregulated in response to mitochondrial dysfunction and excessive reactive oxygen species (ROS) production. We found a significant reduction in maximal mitochondrial respiration capacity (i.e., oxygen consumption rate [OCR]) in IM muscle compared to CTRL (− 25%; p < 0.05; Fig. 3A). This decrease was accompanied by elevated mitochondrial hydrogen peroxide (mH_2_O_2_) production when stimulated by succinate (+ 48%; p < 0.05; Fig. 3B). A similar trend was observed with pyruvate-malate-succinate stimulation of mH_2_O_2_, although the increase was not statistically significant (+36%; p=0.07; Fig. 3C). Additionally, electron leak was increased (+ 66%; p < 0.05; Fig. 3D), which is indicative of inefficient electron transport, without modifying oxidative phosphorylation (OXPHOS) coupling (Fig. 3E). Together, these findings suggest that IM induces substantial impairments in mitochondrial function.

### Mitochondrial localization of BNIP3 and activation of intrinsic apoptotic signaling define immobilization-induced atrophy

We found that the autophagic cargo protein SQSTM1 was reduced (−46%; p < 0.05), while the autophagy recognition protein LC3B showed little variation, suggesting a differential regulation of autophagic components in response to immobilization (Fig. 3F, G). In addition, lipid peroxidation was increased in IM muscle, as evidenced by a 50% increase in 4-hydroxynonenal (4HNE) (Fig. 3F, G), which coincided with increased mH_2_O_2_ emission (Fig. 3B). At the mitochondrial level, there was a noticeable reduction in lipidated LC3B-II and SQSTM1 in IM muscle (Fig. 3F, H), whereas only SQSTM1 was reduced in RM muscle (Fig. 3F, H). Conversely, mitochondrial localization of the mitophagy receptors BNIP3, FUNDC1, and BCL2L13 were significantly elevated in both IM and RM muscle (Fig. 3F, H), suggesting a persistent activation of mitophagy. However, no significant changes were observed in other mitophagy-related proteins including mitochondrial levels of PINK1 and PRKN (Fig. 3F, H).

Considering the RNA-Seq analysis (using a publicly available dataset) showing an upregulation of genes linked to intrinsic apoptotic signaling in muscle tissues following IM (Fig. 1D), we examined the involvement of key regulators of mitochondrial outer membrane permeabilization (MOMP). Our analysis revealed no significant changes in the mitochondrial localization of the pro-apoptotic protein BAX or the anti-apoptotic protein BCL2 (Fig. 3F, I). However, an increased BAX/BCL2 ratio was detected in RM muscle, indicative of increased mitochondrial apoptotic signaling (Fig. 3I). Further exploration into the downstream signals of intrinsic apoptotic pathways showed 20–30% elevation in caspase 9 (CASP9) and caspase 3 (CASP3) activity in both IM and RM muscle compared to CTRL (Fig. 3J). Additionally, our results indicated a suppression of calpain (CAPN) activity in IM muscle (− 44%; p < 0.05; Fig. 3J). In contrast, CAPN activity was significantly higher in RM muscle (+ 85%; p < 0.05). Collectively, these findings illustrate that immobilization-induced muscle atrophy not only impaired mitochondrial function and heightened mitophagy but also modified mitochondrial-specific apoptotic and proteolytic signaling pathways.

### Immobilization suppresses mitochondrial biogenesis and fusion signals, while upregulating key mitophagy molecules

**Fig. 4:**
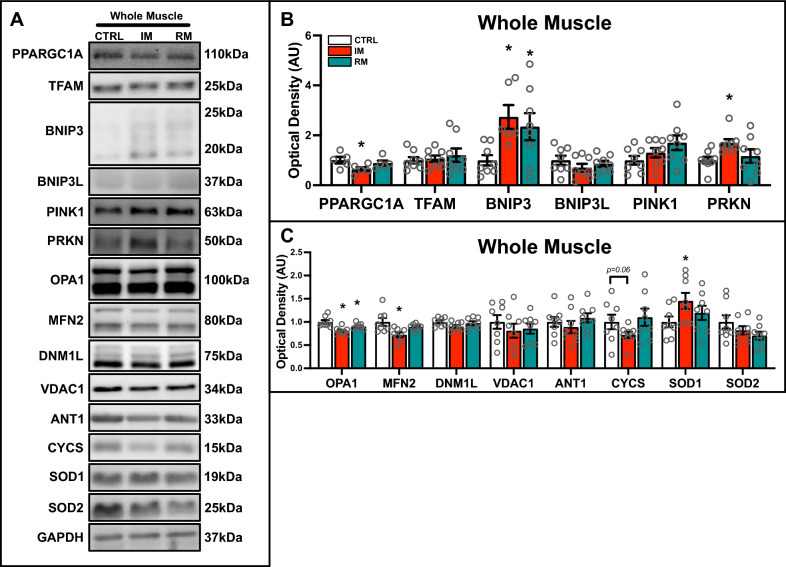
Quantitative analysis of mitochondrial remodeling proteins in IM and RM muscle. **A** Representative immunoblots from whole muscle lysates. **B** Quantitative analysis of PPARGC1A, TFAM, BNIP3, BNIP3L, PINK1, and PRKN. **C** Quantification of OPA1, MFN2, DNM1L, VDAC1, ANT1, CYCS, SOD1, and SOD2 in whole muscle lysate. * p < 0.05 compared to CTRL group. n = 8 per group

IM led to a reduction in mitochondrial biogenesis and fusion proteins, including PPARGC1A (also known as PGC-1α; −28%; p < 0.05), OPA1 (−20%; p < 0.05), and MFN2 (−28%; p < 0.05; Fig. 4A, B). Notably, PPARGC1A and MFN2 returned to baseline upon remobilization; however, OPA1 remained depressed (−10%; p < 0.05). Additionally, IM upregulated the mitophagy-associated proteins BNIP3 (+ 170%; p < 0.05) and PRKN (+ 70%; p < 0.05), without modifying PINK1 or BNIP3L (Fig. 4A, B). Importantly, BNIP3 remained elevated following RM (+ 130%; p < 0.05), while PRKN was restored, suggesting differential regulation of mitophagy during the recovery process. Moreover, the mitochondrial biogenesis marker TFAM (Fig. 4A, B), the fission protein dynamin-1-like protein (DNM1L) (Fig. 4A, C), and various mitochondrial-related proteins including voltage-dependent anion channel 1 (VDAC1), adenine nucleotide translocator 1 (ANT1), cytochrome c (CYCS), and superoxide dismutase 2 (SOD2) only showed non-significant fluctuations (Fig. 4A, C). Taken together, our findings demonstrate that IM induces significant physiological adaptations at the myofiber and mitochondrial levels.

### NAC treatment during immobilization suppresses mitophagic flux and exacerbates atrophy of type IIX and IIB fibers

**Fig. 5:**
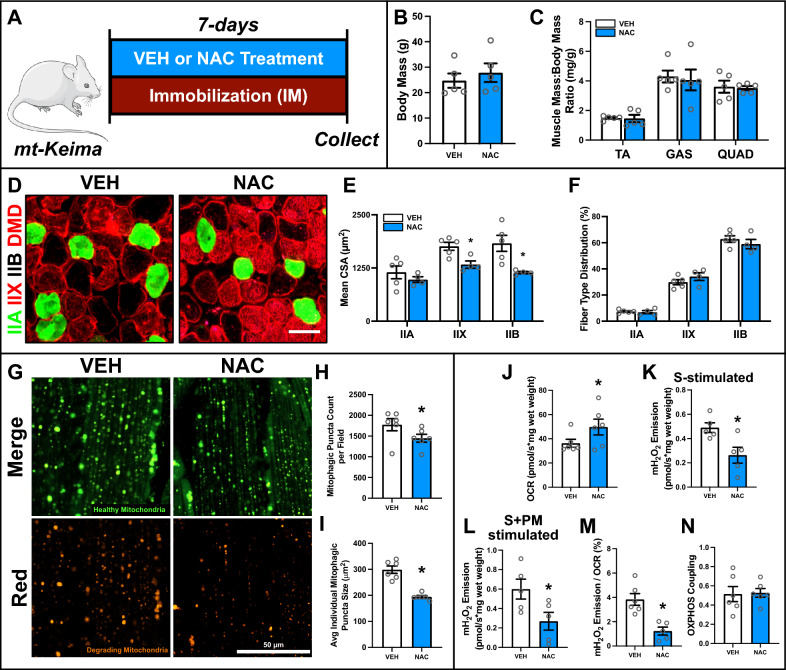
Effect of N-acetylcysteine (NAC) on IM muscle. **A** Representative visualization of IM and NAC treatment protocol. **B** Body mass of vehicle (VEH) and NAC treated mice. **C** Skeletal muscle mass of TA, GAS, and QUAD normalized to body mass. **D** Representative immunofluorescent images of muscle from VEH and NAC treated IM mice. Samples were stained to identify myosin heavy chain isoform and fiber membrane: type IIA (green), type IIX (red), type IIB (unstained), and dystrophin (DMD, red). Scale bar indicates 50 μm. **E** Quantification of mean CSA and **F** distribution of type IIA, IIX, and IIB fibers. **G** Representative confocal images of mitophagic flux from muscle of IM mt-Keima reporter mice treated with VEH or NAC. Green fluorescence indicates healthy mitochondria. Red fluorescence indicates degrading mitochondria. Scale bar indicates 50 μm. Quantification of **H** mitophagic (i.e., red) puncta count per field and **I** average individual mitophagic puncta size. **J** Quantification of maximal oxygen consumption rate (i.e., respiration) of permeabilized bundles. Simultaneous quantification of **K** succinate-stimulated mitochondrial (mH_2_O_2_) production, and **L** succinate + pyruvate/malate-stimulated mH_2_O_2_ production in permeabilized TA muscle bundles. Quantification of **M** mitochondrial fraction of electron leak and **N** OXPHOS coupling. * p < 0.05 compared to VEH group. n = 5–8 per group

Given the observed impaired mitochondrial function, activation of mitophagy, mitochondrial CASP signaling, and oxidative stress, we hypothesized that antioxidant treatment could mitigate these detrimental effects in IM muscle. Contrary to our expectations, N-acetylcysteine (NAC) treatment did not modify body mass nor preserve relative TA, GAS, and QUAD mass (Fig. 5A–C). Surprisingly, NAC treatment during IM led to even greater reductions in the mean CSA of type IIX (−24%; p < 0.05) and IIB (−39%; p < 0.05) fibers, but did not affect fiber type distribution (Fig. 5D–F). Furthermore, NAC treatment significantly attenuated mitophagy, as shown by reduced mitophagic puncta count (−20%; p < 0.05) and smaller average individual puncta size (− 33%; p < 0.05; Fig. 5G–I). These findings suggest that antioxidant treatment suppresses mitophagy while detrimentally impacting fiber atrophy.

**Fig. 6:**
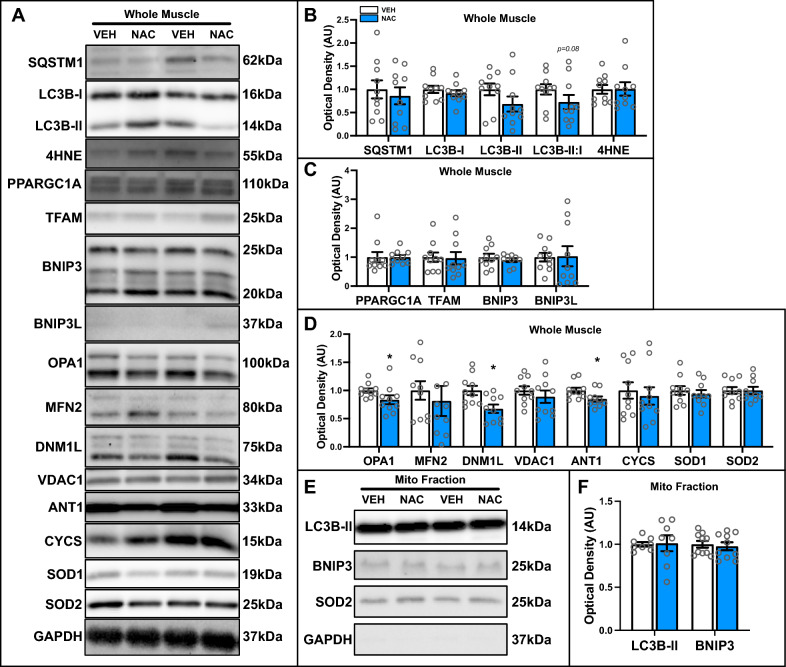
Quantitative analysis of autophagy and mitophagy proteins in VEH and NAC treated IM muscle. **A** Representative immunoblots of whole muscle lysates. GAPDH shown as a loading control for whole muscle lysates. **B** Quantitative analysis of SQSTM1, LC3B-I, LC3B-II, LC3B-II:I, and 4HNE from whole muscle lysates. **C** Quantitative analysis of PPARGC1A, TFAM, BNIP3, and BNIP3L from whole muscle lysates. **D** Quantitative analysis of OPA1, MFN2, DNM1L, VDAC1, ANT1, CYCS, SOD1, and SOD2 from whole muscle lysates. **E** Representative immunoblots of mitochondrial fractions. GAPDH (cytosolic) and SOD2 (mitochondrial) shown to confirm purity of mitochondrial fraction. **F** Quantitative analysis of LC3B-II and BNIP3 from mitochondrial-enriched fractions. * p < 0.05 compared to VEH group. n = 5–10 per group

Using high-resolution respirometry, we observed improved mitochondrial function as shown by an increase in maximal mitochondrial respiration (+ 36%; p < 0.05; Fig. 5J), reduced mH_2_O_2_ emission (− 44%; p < 0.05; Fig. 5K, L), and lower fraction of electron leak from NAC-treated IM muscle compared to CTRL (−70%; p < 0.05; Fig. 5M), without changes in overall OXPHOS coupling (p > 0.05; Fig. 5N). There were no differences in whole muscle SQSTM1, LC3B-I, LC3B-II, and 4HNE, but a non-significant (p = 0.08) reduction in the LC3B-II:I ratio (Fig. 6A, B). Further, whole muscle PPARGC1A, TFAM, BNIP3 and BNIP3L were not affected by NAC (Fig. 6A, C). However, OPA1 (−17%; p < 0.05) and DNM1L (−33%; p < 0.05) were reduced, while MFN2 remained unchanged (Fig. 6A, D); suggesting a disruption of mitochondrial fusion and fission processes. Moreover, with the exception of ANT1 (−16%; p < 0.05), NAC treatment did not affect VDAC1, CYCS, SOD1, or SOD2 (Fig. 6A, D). Additionally, no observable differences in the mitochondrial localization of LC3B-II or BNIP3 were detected following NAC treatment (Fig. 6E, F). Together, these results indicate that NAC positively influence mitochondrial function during IM, but exacerbated fiber atrophy likely by limiting mitophagic flux.

### COL treatment during immobilization inhibits mitophagic flux and exacerbates atrophy of type IIX and IIB fibers

**Fig. 7:**
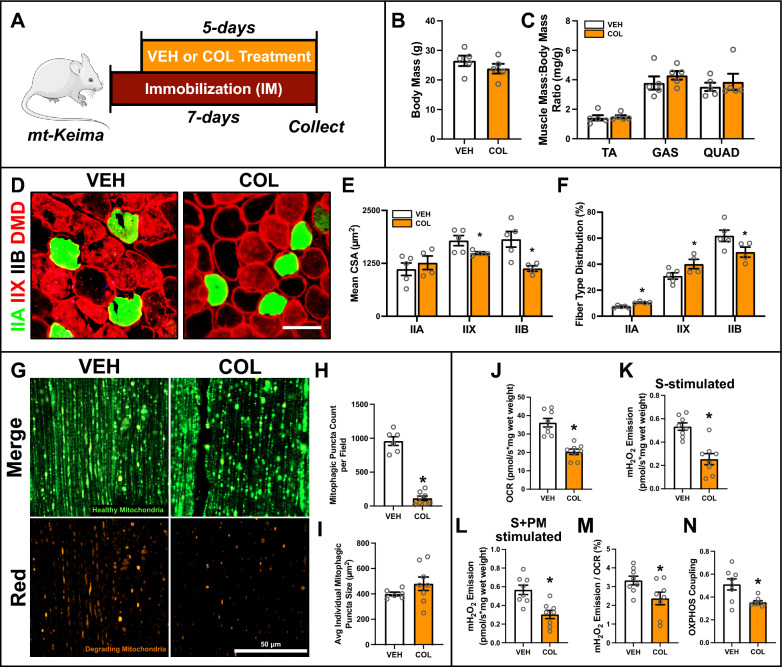
Effect of colchicine (COL) treatment on IM muscle. **A** Representative visualization of IM and COL treatment protocol. **B** Body mass of VEH and COL treated mice. **C** Skeletal muscle mass of TA, GAS, and QUAD normalized to body mass. **D** Representative immunofluorescent images of muscle from VEH and NAC treated IM mice. Samples were stained to identify myosin heavy chain isoform and fiber membrane: type IIA (green), type IIX (red), type IIB (unstained), and dystrophin (DMD, red). Scale bar indicates 50 μm. Quantification of **E** mean CSA and **F** distribution of type IIA, IIX, and IIB fibers. **G** Representative confocal images of mitophagic flux from muscle of IM mt-Keima reporter mice treated with VEH or NAC. Green fluorescence indicates healthy mitochondria. Red fluorescence indicates degrading mitochondria. Scale bar indicates 50 μm. Quantification of **H** mitophagic (i.e., red) puncta count per field and **I** average individual mitophagic puncta size. **J** Quantification of maximal oxygen consumption rate (i.e., respiration) of permeabilized bundles. Simultaneous quantification of **K** succinate-stimulated mitochondrial (mH_2_O_2_) production, and **L** succinate + pyruvate/malate-stimulated mH_2_O_2_ production in permeabilized muscle bundles. Quantification of (**M**) mitochondrial fraction of electron leak and **N** OXPHOS coupling. * p < 0.05 compared to VEH group. n = 4–8 per group

Given the above findings we explored the effects of pharmacologically inhibiting mitophagy on muscle and mitochondrial parameters following IM. Due to the absence of specific inhibitors of mitophagy, we utilized colchicine (COL), a known inhibitor of microtubule polymerization, to broadly inhibit the autophagy machinery. COL treatment of IM mice did not modify body mass or normalized TA, GAS and QUAD mass (Fig. 7A–C). However, CSA of type IIX (− 12%; p < 0.05) and IIB (− 40%; p < 0.05) fibers were reduced (Fig. 7D, E). Interestingly, we also noted a preferential loss of type IIB fibers, along with a compensatory increase in type IIA and IIX fiber distribution (Fig. 7F). Furthermore, COL suppressed mitophagic flux as evidenced by a significant reduction in mitophagic puncta count (−88%; p < 0.05), but did not affect average individual puncta size (Fig. 7G–I). These findings highlight that inhibition of the autophagy machinery via COL treatment limits mitophagic flux and leads to greater muscle atrophy in mice following IM. COL also caused marked mitochondrial dysfunction, as evidenced by decreased maximal respiration (− 45%; p < 0.05; Fig. 7J), mH_2_O_2_ emission (− 53%; p < 0.05; Fig. 7K-L), electron leak − 31%; p < 0.05; Fig. 7M), and impaired OXPHOS coupling efficiency (− 30%; p < 0.05; Fig. 7N).

**Fig. 8:**
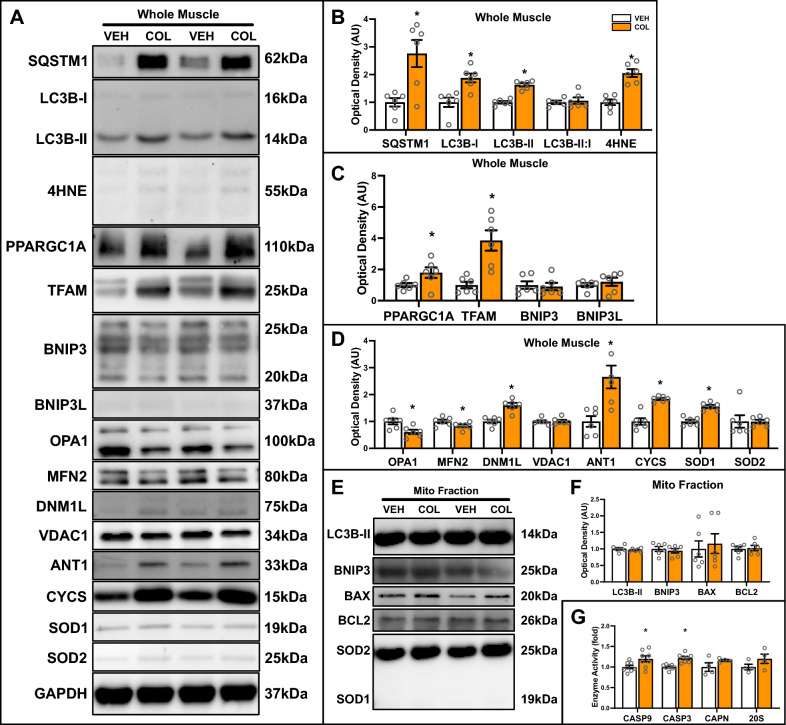
Quantitative analysis of autophagy and mitophagy proteins in VEH and COL treated IM muscle. **A** Representative immunoblots of whole muscle lysates. GAPDH shown as a loading control for whole muscle lysates. **B** Quantitative analysis of SQSTM1, LC3B-I, LC3B-II, LC3B-II:I, and 4HNE from whole muscle lysates. **C** Quantitative analysis of PPARGC1A, TFAM, BNIP3, and BNIP3L from whole muscle lysates. **D** Quantitative analysis of OPA1, MFN2, DNM1L, VDAC1, ANT1, CYCS, SOD1, and SOD2 from whole muscle lysates. **E** Representative immunoblots of mitochondrial fractions. SOD1 (cytosolic) and SOD2 (mitochondrial) shown to confirm purity of mitochondrial fraction. **F** Quantitative analysis of LC3B-II, BNIP3, BAX and BCL2 from mitochondrial-enriched fractions. **G** Quantitative analysis of CASP9 activity, CASP3 activity, CAPN activity, and 20S proteasome activity. * p < 0.05 compared to VEH group. n = 6–8 per group

Immunoblot analyses further supported these findings, as there was an accumulation of SQSTM1 (+ 170%; p < 0.05), LC3B-I (+ 87%; p < 0.05), LC3B-II (+ 63%; p < 0.05), and 4HNE (+ 100%; p < 0.05) (Fig. 8A, B). COL treatment led to elevated levels of mitochondrial biogenesis and other mitochondrial markers, including total PPARGC1A, TFAM, DNM1L, CYCS, ANT1, and SOD1 (Fig. 8A, C, D). This suggests a compensatory response, potentially due to reduced degradation of these proteins. Conversely, the mitochondrial fusion proteins OPA1 and MFN2 were suppressed in IM muscle treated with COL (Fig. 8A, D). Despite this, mitochondrial LC3B-II, BNIP3, BAX, and BCL2 were not altered (Fig. 8E-F). However, CASP9 (+ 19%; p < 0.05) and CASP3 (+ 21%; p < 0.05) activity increased in COL treated mice, but not CAPN activity nor 20S proteasome activity (Fig. 8G). Taken together, these findings indicate that COL-induced inhibition of mitophagy exacerbates mitochondrial dysfunction and oxidative stress, and specifically activates mitochondrial apoptotic signaling.

## Discussion

Skeletal muscle disuse is characterized by a significant loss of muscle mass and strength. Recent work has implicated mitochondrial remodeling, particularly mitophagy, in disuse atrophy [3, 16, 32], though the exact mechanisms and role of mitophagy in the atrophic process remain unclear. In the present study, we found that: (1) immobilization-induced atrophy is accompanied by mitochondrial localization of multiple mitophagy receptors and enhanced mitophagic flux, (2) antioxidant treatment of immobilized mice partially reduced mitophagy and caused greater fiber atrophy, and (3) inhibition of the autophagic machinery and mitophagic flux negatively impacts mitochondrial function that was accompanied by exacerbated fiber atrophy.

### Impact of hindlimb immobilization on mitochondrial remodeling

Immobilization-induced skeletal muscle atrophy results in significant structural remodeling, characterized by the extensive degradation of contractile filaments and other cellular components [34, 35]. This structural remodeling is primarily attributed to a molecular shift from growth-promoting to degradation-signaling pathways [34]. Increased expression of proteolytic genes such as tripartite motif containing 63 (*Trim63)* and F-box protein 32 (*Fbxo32)* [17, 36–38] and activation of other degradative pathways like apoptosis are characteristic of disuse atrophy [20, 39–41]. In fact, proteomic analyses have highlighted significant mitochondrial remodeling in IM human vastus lateralis muscle, showing downregulation of genes related to mitochondrial organization and metabolism, and upregulation of genes linked to apoptosis and ubiquitination [42]. Our RNA sequencing analysis aligns with these findings, showing increased DEGs associated with cellular stress (UPS and apoptosis) and decreased DEGs related to muscle contraction and mitochondrial function. Gene ontology analyses confirm these patterns, reinforcing the universal mechanisms driving skeletal muscle atrophy and the relevance of our rodent model to human muscle atrophy.

Our RNA sequencing results are bolstered by our in vivo experiments that illustrate progressive skeletal muscle loss in IM mice, with partial recovery following RM. Notably, 7-days of IM led to considerable skeletal muscle loss and weakness, coupled with increased mitophagic flux, which corroborate prior findings [16]. This increase in mitophagy aligns with other disuse atrophy models like denervation, where flux is enhanced within 24 h [3]. Our data also indicate fiber type-specific responses, with type IIX and IIB fibers undergoing significant atrophy without full recovery following RM, whereas type IIA fibers regain their original size. Our previous work found that oxidative muscle exhibits lower autophagic flux [26, 43], potentially moderating the extent of remodeling in response to atrophic stimuli like IM. Overall, our findings confirm a consistent link between skeletal muscle atrophy and mitophagic flux during IM and RM.

We observed mitochondrial dysfunction characterized by reduced maximal respiration, increased mH_2_O_2_ emission, and higher electron leak in IM muscle. These changes occurred along with elevated whole muscle lipid peroxidation and are consistent with previous findings in disuse atrophy models [3, 44–47]. Importantly, the negative alterations resulting from IM were accompanied by increased mitophagic flux, as evidenced by greater mitophagic puncta count. The elevated mitophagic flux may be a compensatory response to mitigate further myofiber damage. Although changes in autophagy markers were not pronounced in whole muscle or mitochondrial fractions, mitophagic flux experiments indicated active and increased mitochondrial degradation. We noted significant mitochondrial localization of BNIP3, FUNDC1, and BCL2L13 in IM mice that also persisted after 7-days of RM, consistent with previous findings [15, 32]. While mitophagy-related molecules and their transcripts have been found to be elevated in IM muscle [14–16], this is the first report of elevated BNIP3, FUNDC1, and BCL2L13 in the mitochondrial fraction. These molecules may influence several mitochondrial parameters, including bioenergetics, mitophagic flux, and apoptosis [48–54]. The translocation of these molecules to the mitochondria may result from the mild hypoxic effects caused by the gentle constriction of hindlimb taping, which could also reduce blood flow [55–57]. Hypoxia-inducible factor 1 subunit alpha (HIF1A) is a key regulator of the cellular response to hypoxia and may be activated in this model by localized oxygen deprivation resulting from reduced blood flow. Under these conditions, HIF1A stabilization occurs due to the inhibition of prolyl hydroxylases, which normally target HIF1A for degradation [58]. This stabilization allows HIF1A to translocate to the nucleus, where it drives the transcription of genes associated with the hypoxic stress response, including *BNIP3*, *FUNDC1*, and *BCL2L13* [59–61]. Nonetheless, as we explored other BH3-containing molecules, we observed increased BAX:BCL2 ratio in mitochondrial fractions from RM muscle suggesting MOMP formation, with elevated CASP9 and CASP3 activity in IM and RM muscle further confirming the role of mitochondrial apoptotic signaling in skeletal muscle atrophy and dysfunction.

Augmented activation of degradative pathways are linked to significant changes in molecular signaling, including the suppression of PPARGC1A, the master regulator of mitochondrial biogenesis, in IM muscle [14, 62, 63]. Our findings confirm the reduction of PPARGC1A, likely due to decreased energy demands in IM muscle. Furthermore, changes in mitochondrial dynamics have been observed, with some studies noting increased DNM1L, indicative of mitochondrial fission or fragmentation following disuse [64–66], although this has not been universally observed [33, 46]. Our data show no change in DNM1L, but decreased OPA1 and MFN2 in IM muscle, suggesting a shift towards fragmentation. This shift may occur early, as increases in DNM1L have been detected shortly after disuse onset, alongside reduced *Opa1* and *Mfn1/2* transcripts [62, 64]. The elevated mitophagic flux in IM muscle in this study suggests that mitochondrial dynamics may shift towards fission early to facilitate efficient degradation.

### NAC treatment suppresses mitophagy flux and worsens immobilization-induced muscle atrophy

Given the expansive literature regarding a role of ROS during skeletal muscle atrophy, we explored modulating ROS during IM with NAC. We found that NAC did not preserve skeletal muscle mass, which is consistent with studies showing limited antioxidant efficacy [46, 67]. However, other potent antioxidants like vitamin E and mitochondrial-targeted antioxidants have shown partial or complete skeletal muscle mass preservation [68–71]. Interestingly, NAC treatment led to selective atrophy in type IIX and IIB fibers, along with reduced mitophagic flux, marking the first report of such fiber type specific atrophy in IM muscle. Type IIX and IIB fibers in mouse TA constitute about 90% of total fibers and displayed an additional 24% to 39% reduction in CSA following NAC administration compared to VEH. Previous studies have highlighted the role of ROS emission as a molecular trigger for mitophagy [72–75]. Notably, the inhibition of ROS through antioxidant treatment (e.g., NAC, SOD-mimetic, catalase, MitoQ, etc.) consistently led to suppressed mitophagy across these studies [72–75]. In specific cell types, such as human cardiomyocytes but not human fibroblasts, the inhibition of mitophagy even resulted in the accumulation of defective mitochondria [75]. These findings suggest that ROS is essential for mitophagy, particularly under stress conditions, and ROS may also play a critical role in regulating the mitochondrial pool in cells or tissues with higher metabolic demands. In our hands, NAC improved mitochondrial respiration and reduced ROS emission; however, it did not alter mitochondrial localization of LC3B-II and BNIP3. Our observations indicate that while NAC limits mitophagic flux, it does not affect the localization of autophagic and mitophagic proteins at the mitochondrial level. It is possible that alterations in the redox state induced by NAC might interfere with the molecular interactions necessary for cargo recognition at the mitochondrial level, such as the dimerization of BNIP3 at the mitochondria by preventing disulfide bond [76]. Additionally, NAC treatment may impair the autophagy machinery by limiting the phosphorylation of upstream molecules like mitogen-activated protein kinases (MAPK) and BCL2 [77, 78]. Moreover, these findings indicate that ROS signaling is essential for mitophagy, while reducing ROS signaling suppresses mitophagic flux and exacerbates muscle atrophy in IM muscle.

### COL treatment disrupts mitochondrial proteostasis, activates CASP signaling, and contributes to greater atrophy of immobilized muscle

Our data show that skeletal muscle atrophy is accompanied by increased mitophagy. Although NAC treatment suppresses mitophagy and improves mitochondrial function, it not only did not prevent the loss of muscle mass but exacerbated this phenotype. To explore further, we treated IM mice with COL, which has been used extensively to inhibit autophagy/mitophagy in skeletal muscle [4, 26, 79]. These studies found that COL treatment led to an accumulation of total and mitochondrial-localized LC3B-II, SQSTM1, and PRKN, indicating a disruption in mitochondrial targeting and a halt in mitophagic flux [4, 26, 79]. To complement these earlier studies, we observed reduced mitophagic flux following extended COL treatment in IM muscle. Furthermore, COL led to even greater atrophy of type IIX and IIB fibers during IM and a shift toward a slower contractile phenotype, with more type IIA and IIX fibers and fewer type IIB fibers. Previous research found autophagy-deficiency exacerbates skeletal muscle loss in denervation models by disrupting proteostasis and accumulating protein aggregates including SQSTM1 and ubiquitin [10]. While denervation causes more severe skeletal muscle loss than immobilization-induced atrophy [3, 10, 33], our results primarily show selective atrophy in fast/glycolytic fibers. Given that fast/glycolytic muscle is particularly vulnerable to autophagy deficiency [26, 43], we propose that the reduced CSA of type IIX and IIB fibers and the shift in fiber type distribution are adaptive responses to compromised autophagic and mitophagic signaling in these fibers. COL treatment of IM mice significantly impaired mitochondrial function, as evidenced by reduced maximal respiration, decreased coupling efficiency, and increased whole muscle lipid peroxidation. Interestingly, we also observed reduced mitochondrial ROS emission, consistent with previous studies that demonstrated an inhibitory effect of COL on ROS production in vitro [80–82]. Our findings also align with prior findings of compromised mitochondrial function from the lateral quadriceps of mice treated for 2-days with COL [83].

We also observed an accumulation of SQSTM1, LC3B-I, and LC3B-II in whole muscle lysates with COL which is similar to previous reports with 1–2 days of COL [3, 4, 26, 79, 84]. Although we did not observe any differences in the mitochondrial localization of autophagy or mitophagy-related proteins with COL, this is consistent with its role as a microtubule inhibitor, which can limit the translocation of cytosolic proteins [85–87]. We hypothesize that prolonged inhibition of microtubule polymerization (i.e., 5-days) allows for the tagging of mitochondria by mitophagy-related proteins like BNIP3, but restricts efficient targeting by autophagosomes, resulting in inadequate mitochondrial removal. Nonetheless, BNIP3 has previously been shown to be induced in autophagy-deficient models, along with CASP activation and initiating mitochondrial apoptotic signaling [10]. While mitochondrial localization of apoptotic molecules (i.e., BAX) remained unchanged in COL treated mice, we observed elevated CASP9 and CASP3 activity, indicating the activation of mitochondrial apoptotic stress responses in an autophagy-deficient state. This phenomenon has been previously documented in vitro by our group [88, 89]. Taken together, treatment of IM mice with COL confirmed that inhibiting mitophagy triggers several detrimental effects, including mitochondrial dysfunction and the activation of mitochondrial apoptotic signaling, which collectively contribute to the exacerbation of muscle atrophy. These findings align with our earlier data showing that NAC treatment also exacerbated muscle atrophy in IM muscle by suppressing mitophagy.

### Mitophagy protects against immobilization-induced muscle atrophy

Mitophagy plays an essential role in mitigating the deleterious effects of mitochondrial damage. This is particularly vital in post-mitotic cells, such as skeletal muscle cells/fibers that accumulate cellular damage over time. The significance of mitophagy extends to various skeletal muscle-specific conditions, including denervation, spinal cord injuries, muscular dystrophies, and aging [2, 90]. In the case of disuse atrophy, increased autophagy and mitophagy signaling along with heightened mitophagic flux have been reported [3, 4, 16, 32]. The current study questioned whether mitophagy acts as a protective mechanism against muscle atrophy or contributes to greater atrophy by reducing the overall mitochondrial pool. Our findings reveal that suppression of mitophagy through antioxidant administration or COL exacerbated IM-induced atrophy in glycolytic fibers, specifically type IIX and IIB. Despite the differing impacts of NAC and COL on mitochondrial function, the end result was a similar degree of muscle atrophy. This observation highlights the importance of sustaining mitophagic activity for preserving muscle integrity, indicating that this process is crucial regardless of the initial mitochondrial condition. Our findings are consistent with previous work showing that *Atg7*-deficient TA muscle experienced significantly greater atrophy after denervation compared to controls, along with abnormal mitochondrial structure [10]. Interestingly, enhancing mitophagic flux with treatments like urolithin A in rodents has been shown to improve muscle function, as evidenced by increased grip strength, maximal running distance, upregulated mitophagy-related genes (i.e., *Bnip3*, *Pink1*, *Prkn*, *Becn1*), and increased CSA in *mdx* mouse muscle [12, 13]. Randomized clinical trials in humans supplementing urolithin A have found elevated markers of mitochondrial function, PRKN levels, and overall muscle performance [91–94]. These findings underscore the potential of promoting mitophagy to counteract skeletal muscle atrophy in a variety of conditions, and further emphasizing the need for ongoing research. Overall, our results confirm the necessity of mitophagy in limiting atrophy during disuse.

## Conclusion

In conclusion, this study provides new insights into the molecular and physiological adaptations linked to immobilization-induced skeletal muscle atrophy. Our findings reveal that mitochondrial dysfunction and increased mitophagic activity are key features of immobilization-induced atrophy. We also demonstrate that mitophagy acts as a protective mechanism against excessive muscle loss, as interventions that reduce mitophagic flux, either through antioxidant treatment or pharmacological inhibition of microtubule polymerization, exacerbate skeletal muscle atrophy. These results highlight the critical role of balanced mitochondrial quality control in maintaining muscle mass during disuse. Future research should explore various aspects of mitochondrial remodeling, particularly mitophagy, in disuse atrophy. This may lead to targeted therapies that prevent muscle loss without compromising cellular function.

## Data Availability

The datasets during and/or analysed during the current study available from the corresponding author on reasonable request.

## Abbreviations

ANT1: Adenosine nucleotide translocator 1
ATG7: Autophagy related 7
BNIP3: BCL2 interacting protein 3
BNIP3L: BNIP3 like
CAPN: Calpain
CASP: Caspase
COL: Colchicine
CYCS: Cytochrome c
DNM1L: Dynamin 1 like
GAS: Gastrocnemius
HIF1A: Hypoxia inducible factor 1 subunit alpha
MAP1LC3B/LC3B: Microtubule associated protein 1 light chain 3 beta
MFN2: Mitofusin 2
NAC: N-acetylcysteine
OPA1: Optic atrophy 1
PINK1: PTEN induced kinase 1
PPARGC1A: PPARG coactivating 1 alpha
PRKN: Parkin
QUAD: Quadriceps
SOD: Superoxidase dismutase
SQSTM1: Sequestosome 1
TA: Tibialis anterior
UPS: Ubiquitin–proteasome system
VDAC1: Voltage dependent anion channel 1
